# Discussion before the Ohio State Dental Society

**Published:** 1873-03

**Authors:** 


### PROCEEDINGS OF SOCIETIES.

### Discussion Before the Ohio State Dental Society Continued.

*Subject—The Best Means of Preserving the Eyesight of Dentists.*

Dr. L. Buffett thought some refrained from the use of glasses to help the eyesight because their patients might think them too old to operate. This is all wrong. As soon as the eyes become deficient glasses should be resorted to. Watch-makers use magnifying glasses all the time, and why not dentists? He felt satisfied glasses would benefit young dentists even.

With regard to preserving the eyesight he said anything that will improve the general health will help the eyesight. Persons of a nervous temperament should not rise too early; lying in bed in the morning, when it is found refreshing, seemed necessary to some people, and he believed we should follow inclination in the matter of sleep.

Dr. R. G. Warner said it was evident to his mind that the nervous and sleepless condition was brought upon people by sitting up late and occupying their minds with study or business. If they would dismiss these things from the mind and go to bed sooner it would be better for them.

Fowler and Wells, he believed, in writing concerning the eyes said, that mush and milk, and temperance, were as good for the eyes as anything else.

Dr. C. B. Kelsey—Does the Doctor mean that mush and milk are good for the eyes?

Dr. W. M. Herriott indorsed Dr. Buffett's remarks on sleep for nervous persons.

It is necessary to keep the stomach in good condition; also to keep the circulation of the blood equal, especially at the extremities. Tight boots in winter often keep the feet cold.

Dentists should have a northern light, and he favored transmission through a single pain, and not through three or four panes. He also favored the use of glasses.

Dr. D. R. Jennings thought that holding the hands before the face is injurious to the operator. The mouth mirror when properly used is of much advantage.

Dr. B. F. Rosson said he had not been ashamed to use glasses for a number of years. But he only used them while operating. He discouraged the use of nose glasses and preferred spectacles.

Dr. J. M. Porter thought the trouble dentists have is of one kind almost universally, because their work is such as produces a certain kind of disease, and because they have no rest in making their operations.

In order to afford the proper relief when operating, the object to relieve the eye must be more than twenty feet from the eye. It may be supposed they may get relief by looking around the room, but in looking at objects at a distance of more than twenty feet the eye is at rest, and there is no such relief in looking at close objects. When looking at a tree or some object at a distance of forty or fifty feet, the eye is at rest, and there is no work for the muscles. When you bring an object near the eyes you have that convergence referred to and an effort on the part of the internal recti muscles, and the nearer you bring the object the more convergence there is of the eyes to see it. Some gentleman spoke of the injury to the eyes, caused by getting them down so close to the patient. That is true, because the nearer the eye is brought to the cavity in the tooth where you are working, the more convergence there is, and the more pressure there must be on the surface of the lens in order to adapt it to this short distance. When looking at an object near you there is a contraction of the ciliary muscles, and the further we get an object from the eye the less strain there is upon these muscles. This thing of the eyeballs enlarging is all bosh. The eyeball is the same when looking at objects at ten feet distant as at fifty feet. When the eyesight begins to quiver you may know that the internal recti muscles are becoming fatigued and weakened, and that rest is necessary. The same principle will apply here as anywhere else in the body. Let a man use a certain set of muscles constantly, and by and by they will become overworked, and disable him from doing what he formerly could do.

The use of the glass as recommended by Dr. Buffett is very good, because by its use less convergence is required, and consequently there is less strain on the eyes.

Another point suggested by another gentleman is very well, that is, that just as soon as you discover that your eyesight is failing, you should resort to glasses; yet it is a better practice to use them before you discover that they are failing. Most any man knows enough to carry an umbrella when it is raining, but it is a pretty good plan to have one along when it does not rain, because it might rain. Eye troubles are more easily anticipated than remedied after they are injured.

The dentist in his operations will have his eye a foot from the cavity which he is filling, next perhaps three feet from the cavity, looking for something he wants, thus using the same muscles of the eye all the time he is at work, and using them the same way day in and day out; and this constant effort to maintain one position, and looking at objects at a certain distance from the eye, causes the eye troubles of which he complains.

Dr. Rosson observed that another advantage from the use of glasses was that it allowed greater distance of the operator from the patient.

Dr. J. H. Warner considered this subject of magnifying glasses one of extreme importance. He commenced the use of magnifying glasses years before he felt any need of it simply because he had one, and believed by its use he had done his eyes an irreparable injury. He believed any one who uses magnifying glasses before he needs them, will by and by come to that point when he can do no better with magnifying glasses than without them. All the mechanical appliances brought to their aid have their proper place in the economy of the profession, but when the eyes become weakened by age or otherwise, then he believed the magnifying glass would be beneficial, but let those whose eyes are strong enough to do the work without that aid use them, and he believed it would be detrimental to them. A recent writer has said that the epizooty, if it were good for nothing else, was good for the dyspepsia, because people who were accustomed to ride will now begin to walk, which will benefit them. He believed this to be true.

The essays read had carefully considered the subject. Dr. Porter's particularly was fraught with all that could be said in the same space of time that was valuable. He had taken a learned view of this subject, and had studied the matter carefully, but theories are sometimes imperfect, and the theory advanced by Dr. Porter, that the constant use of muscles will ultimately end in their destruction, he believed to be untrue. No arm was stronger than the arm of the blacksmith, and he is using the muscles of his arm constantly. It is not the use, but the abuse, of muscles that injures them. He believed the eyes of dentists were injured more by operating by gaslight than many of them were willing to allow. In his own case, however, his eyes had been injured by nothing to so great an extent as by the use of magnifying glasses before he felt the need of them.

Dr. J. M. Porter had hoped that what he had previously said would be understood, but he feared it was not. The point he made was that such eye-troubles as dentists have, were produced by the constant converging position maintained, thereby injuring the internal recti muscles. To compare the action of these muscles with the muscles in the arm of the blacksmith is quite unfair, though he did not accuse the gentleman with intentional unfairness. The blacksmith in using his arm is constantly moving and changing its position, bring into use different muscles; while in the case of the dentist, it is the constant contraction of the internal recti muscles acting against the external recti muscles that causes the injury, and which action is materially different from the constantly changing of the muscles brought into use in the case referred to.

In regard to the use of magnifying glasses, the point is this: do not use magnifying glasses all the time, because if you do, you will have the same trouble as if they were not used at all, as the muscles of the eye would have to be held all the while in the same position. But if the eyes were used half an hour or so without glasses, and then the magnifying glasses used awhile, there would be that change needed, as the amount of convergence necessary to see the same object with the magnifying glass—the object being enlarged—is less than without; it consequently affording relief to the muscles of the eye, as the amount of muscular action required is less. What is wanted is that relief to the muscles doing the work, afforded by frequent change, and one of the best things the operator can do is to operate ten or fifteen minutes with the magnifying glass, and then lay it down, and working about the same length of time without it, when the glass should again be used, thus producing the change of position and consequent relief the eyes need.

Dr. J. H. Warner said he did not intend to criticise Dr. Porter's essay. He would, however, like to know if the muscles of the eye of the dental operator have not frequent change when he is one moment looking in the cavity of the tooth, the next moment looking around for his gold, and so on during the operation, changing the action of the muscles as the blacksmith does in his work. He thought he had made no unfair comparison in the illustration he had made use of, but that his remarks must have been misunderstood.

Dr. H. A. Smith thought there was really no difference of opinion between Dr. Porter and Dr. Warner when rightly understood. He believed the fatigue experienced is more from the mental effort of the operator, and had more to do with the nerves than the muscles. The turning about, looking at different objects, may afford casual relief, but it is that constant effort of looking into the cavity, and bending over with the whole mind concentrated upon the work that fatigues the operator. He did not think magnifying glasses should be resorted to for relief; it results from overtaxing the nervous system, and rest is what is required. The operator should have plenty of good light, and not overwork himself. He had worked until he had been blind temporarily, simply because he had exhausted himself. He did not think it would be well for him to resort to magnifying glasses. If he were long or short-sighted, which was a diseased conditions of the eyes, he should then have lenses; in either case however he would consult an oculist. Unless there is a diseased condition, the eye does not need lenses. This idea of resorting to glasses, and all that sort of thing, is to be done after age has flattened the eye, or after decayed by age, or overworked, or diseased. He thought this bending over and straining position, in which the operator does his work, obviously tended to nervous exhaustion and congestion, and resulted in injury to the eye.

Dr. Siddall wished to emphasize what Dr. Smith had said in regard to the effect of the exhaustion of the system upon the eyes. He had had a spell of fever for four months, and found for sometime, after resuming work, a difficulty with regard to his eyesight. He believed it all originated from his exhausted physical condition. He had experienced the same difficulty at another time, making it clear to him that the difficulty was occasioned by a want of good physical health. He thought there was a liability to injury from the use of glasses, when the trouble arose from overwork.

Dr. F. H. Rehwinkle said it was with some diffidence he opposed some of the remarks made on the subject. As they observed him in the room, they had doubtless noticed that he generally wore glasses; but if he sat down to read or write, he was obliged to remove the glasses, being near-sighted. He could see persons well enough in the room, but was unable to recognize their features without the aid of glasses. He could not use magnifying glasses in his condition, unless he would use them double. He had tried the plan recommended by Dr. Porter, and fancied that perhaps there was some relief in it, but upon further trial, found he had deceived himself, and found he could get along better with his natural sight than with the aid of glasses.

There is a soothing power exerted upon the eye by so many different circumstances, he thought. Perhaps it might be well to mention some of these. As suggested by Dr. Smith, he believed the mental excitement of the operator had much to do with his eye troubles, as, for instance, when there was an apprehension as to the result of the operation. This was the case to a far greater extent before they had the assistance of the rubber dam. The fear he had experienced from danger of large cavities being swamped when operating, had injured his eyes, he thought, more than anything he had experienced since the rubber dam had been brought to their aid.

He found that the atmosphere had a great deal to do with the work of the eyes, especially with those temperaments that were more or less affected by the electricity in the air.

The state of the body had much to do also in regard to it. If, for instance, he was suffering from the effects of constipation, and a tendency of blood to the brain, his eyes became much more easily fatigued than at other times.

Mistakes in diet may result in serious difficulties in like manner, but as soon as its effects pass away the whole trouble disappears.

Again, the concentration of the sight upon a certain point any great length of time, it is true, produces the result stated by Dr. Porter; yet it was not clear in his mind whether it is not the concentration of the mind, the great strain upon the nervous power, that has more to do with it than the simple muscular strain referred to by Dr. Porter, though all he has said, as to the results of this muscular action, he could conceive to be correct.

Dr. Rehwinkle remarked that he had his chair in his operating room covered with green material, and he had found when his eyes were becoming fatigued and painful from the prolonged strain upon them, that if he would take them off of his work, and allow them to rest on that green color, it had a very soothing effect upon them, and seemed to relieve them of the effects of the strain upon them, and after a little while he was able to go on with the work again. He did not know that he could give the philosophy of this; but it was a fact that green, buff, and some other colors had this soothing effect upon the eyes. Dentists, when papering their rooms, should give attention to this fact, and choose neutral colors, neither light or dark, but those that were not hurtful to the eyes.

Heat, he had no doubt, was also hurtful to the eyesight.

With regard to the value of glasses to those whose eyesight had failed from one cause or another he was unable to speak from personal experience, because so far as his case was concerned, he never wore glasses constantly, and perhaps he had an advantage now in his present age, and at a certain focus which he needed for his operations, and could see better probably than a great many others who wore magnifying glasses.

Dr. Porter desired to make an explanation on one point. He had said that the muscular action on the part of the eye when operating was very dissimilar from the muscular effort in the arm of the blacksmith. He said so still, and he wanted it distinctly understood that to see a small object under a distance of twenty feet, requires a converging action of the sight. If that be so, it does not make any difference whether the operator looks at the teeth of the patient, or looks around to pick up his gold, it does not afford any relief. No small object can be seen under a distance of twenty feet without this convergence, and if so, it requires a very great convergence to see small objects under two feet, and the same muscular action is required all the time; and he contended there was no relief as long as the operator was looking at such small objects so long a time, whether it was the teeth, the gold, Hill's stopping, or what it might be, there was no relief unless there was a material change in the distance of these objects from the eye of the operator. If the operator had to reach twenty-five feet to get his gold, there would be a relief to the muscles controlling the sight, but the difference of the distance from the eye to the teeth or the gold affords no relief.

Dr. H. A. Smith desired to know if in turning around to pick up the gold, and to file the metal plate, etc., there was not some relief of that tension of the mind, and also a relief to the muscles of the eye.

Dr. Porter replied that in working on the plate the same muscles are continued in action as in operating in the mouth.

Dr. W. M. Herriott inquired if in holding the eye in the position spoken of, so as to see objects at a certain distance, the muscles were not on a strain similar to that which the muscles of the arm of a blacksmith would be in holding his sledge out at arm's length.

Dr. Porter replied that there was just as much difference as Dr. Herriott suggested.

Dr. J. H. Warner wished to know how Dr. Porter accounted for the relief he said was afforded the eyes by the use of the magnifying glass, as the change of the focus was so small as to produce but little if any change in the action of the recti muscles. The focus of the eye is not changed, but simply the size of the object increased.

He did not believe, that it is the muscular tension that tended to destroy the eyesight half as much as the mental strain, or the nervous strain, one undergoes in protracted dental operations. Any one knows they do not tire half as fast when the operation is going on smoothly as when the saliva begins to flow and he anticlpates difficulty.

Dr. Porter admitted that the focal distance was not changed under the circumstances referred to, but on the other hand the size of the object was materially changed, so that it does not require so much contraction of the muscles as it did to produce the convergence required; in other words, the convexity of the lens of the eye is not increased as much to see the object after it is magnified as before; therefore, there is a relaxing of these muscles, and consequent relief to them.

Dr. C. H. Harroun thought some interesting points on this subject might be brought out by members giving some of their personal experience in regard to the matter. He stated that he had had pretty serious difficulties with his own eyes, caused mainly by excessive labor, and a very strong light, both in the day time and at night; working in a strong light till four or five o'clock, operating during the day, and in his laboratory by gas light until twelve or one o'clock at night, being obliged to do so for want of proper help. The result was in some six months, amaurosis. He was not aware of the injury he was doing his eyes until it was done. He then rested by taking a gun and going West on a hunting tour. Upon returning his eyes seemed restored, but he was advised not to operate without glasses. He had adopted this plan, and for the past six years had used the same glasses. Though out of doors he could see objects apparently as well as ever, yet when operating he had resorted to glasses.

If he had a patient devoid of a proper amount of animal magnetism, thereby drawing from him, there was naturally more wear and tear, and he felt its effects more upon his eyes. If the patient was in full vigor, so that he got a proper supply from him or her, he had no trouble with his eyes. He thought if this matter could be regulated, that the patient coming into the chair would have a proper amount of animal magnetism, the operator would not be so liable to injure his eyes. Dr. J. Taft once prostrated himself by labor of that kind, from the effects of which he did not recover for some months. He found that it was caused by real exhaustion, from want of a proper amount of animal magnetism in the patient. Dr. Harroun also said he had found it the case sometimes that he drew from his patients and exhausted them.

Dr. J. B. Beauman had suffered somewhat with a failure of his eyesight for the last year or two, but had attributed it solely to his growing older. He was still persisting in seeing without glasses; he did not know whether it was a wise course or not. He had read a year or two ago an article in the *Scientific American*, in which the writer stated, if when we found our eyesight beginning to fail we should persist in using the eyes without glass, we could overcome in a great measure the necessity of resorting to the use of glasses, but if we commenced the use of glasses, we would have to use them continually.

He found when he had a long tedious operation to perform, his eyes became weary and weak; when they got rest, they were right again. He believed the great difficulty was that they overtasked the whole physical organization; the eyes partake of weariness the same as the body, and we work too hard and rest too little.

Dr. J. Taft said it is a subject in which every one is interested, and about which every one has thought perhaps, more or less, for when the eyes become wearied by the labor in the operations in which we engage every day, it must come vividly before the mind of every one, "What must become of my eyes?" And especially when the operations in which we engage day by day, depend so much for their proper performance upon the eyes, it becomes a very important matter indeed.

Knowing that the ground has been gone over, and discussed, both by essays and the discussions that have followed, it would seem presumptious in me, who have not heard anything of the views presented, to say anything on the subject. But after all there is a point or two to which I will advert, with the hope that they may not have been presented, because you all know it is very unpleasant to tell a story after it has been once told. Some people can do that, but I cannot; that is, I feel embarrassed in endeavoring to do so.

I will only make a very few remarks, and not detain you many minutes. The first point that occurs to my mind is, that members of the dental profession do not suffer, as a whole, from diseases of the eye more than many people in other occupations. When I look over the profession, and see those who have labored day after day and continuously, I do not see that they suffer more than many other classes of people. We do not find many blind dentists, or many whose eyes indicate disease. I infer therefore that from some cause or other, our occupation is not one—as it is practised by most dentists—very wearing upon the eyes. Though that is said very frequently, nevertheless, those who operate frequently—especially in the manner common now—experience little injury to the eyes.

The structure of the eye has doubtless been presented here far better than I could do it, and the mode of its operations, the changes it undergoes, and the various labors in which it is occupied, have doubtless also been presented, as has likewise the influence of these operations, the phases of these changes made in the eyes, because these are obvious things. Now if all these have been presented, what is there left for me to discuss.

One thing talked about considerably last night, as you will remember, was the condition of the muscles that move and manage, and hold the eye apparatus. And in the main the correct ideas were presented. It is important in any kind of exercise that we have rest and labor, that there be vicissitudes or changes in this respect. The results obtained by various movements are very different from those attained by the condition of tension. Now the eye to be moved frequently in its socket exercises the muscles of the eye, and of course that will give rest, as was clearly presented last night. The point I would present is this: that every little movement of the muscles of the eye will give it rest, while a strained fixed position will become wearisome. This is shown by the fact that looking at an object of considerable size the eye is not wearied, but if you fix the eye upon a small point at the same distance, and hold it there, and maintain that fixed position on the small point for a considerable length of time, the eye becomes weary. While fixed upon the large object for an equal length of time there will not be that weariness, because there is room for the movement of some portions of the muscles. The great difficulty arises from fixing the eye for a great length of time on a very small point, and holding it there.

The character of the light thrown into the eye also has very much to do with it, not however with respect to the muscles of the eye, but in regard to the influence particular rays of light have upon the organs of sight, or upon the nerve distribution that receives the impression. That was hinted at last night, when some one—I have forgotten who—spoke of the soothing effect of the green rays brought to bear upon the eyes, in which case a portion of the rays of light falling upon the eye are arrested, and only a certain portion strikes the eye. All understand this, that any nerves that receive impressions will receive some that are kind or pleasant, but will not tolerate others. That is true of the eye; some colors are agreeable and pleasant to the eye, while others fatigue or tire it, or irritate it. That is a point I think we should keep in mind, and study. In operating there is just this difficulty sometimes: if the gold which is being manipulated and placed in the cavity is very brilliant, the eye is wearied sometimes from the reflection of this metallic surface, and longs to look at something which will not weary it. Of course, taking the eye from this bright surface will afford relief, but in prolonged operations where the eye is necessarily fixed upon something of this kind for a long time it becomes exceedingly fatigued.

The discussion last night ran largely in reference to the motor, or muscles that manage and turn the eye. In this connection it might be remarked, that the nerves that are distributed through the eye itself may be impinged upon, or encroached upon, so as to injure it very much, and cause the eye to become easily fatigued. It is well known that the nerves become impressed by various influences that operate upon them. The nerves that supply the ear become irritated. In many instances sounds become exceedingly unpleasant and grating, so as to produce irritation upon the nerves of the ear, while other sounds may appear pleasant, soothing, and quieting. So with the sense of smell, the sense of touch, and all the other senses are liable to this hyperæsthetic state. Perhaps we too much overlook this matter. This disposition may occur in various ways. The general enfeeblement of the system will bring about an unfavorable condition of the nerves that may produce hyperæsthesia, or obtund the nerves. The sensibility of all the nerves of the body may be thus increased, and made more susceptible to impressions, or may be obtunded; and it is important to know the facts in regard to this, and be able to understand, and decide whether there is a hyperæsthetic state of, the organ in its nerve distribution, or whethere there is an obtunded condition.

The details of this of course I do not pretend to present here, and perhaps would not be able to present them, but these are points we ought to understand. The eyes are always weaker when there is a general enfeeblement; the muscles that move and manage the eye partake of this enfeebled condition, and become more easily wearied. Those of you who have looked into a camera for two or three minutes, or half a minute perhaps, found your eyes sensibly relieved as soon as the camera was closed up. You scarcely ever see any one with eyes so strong as to be able to look steadlly upon one point for a considerable length of time without fatigue. It is a wonder, that the eyes of dentists do not suffer more than they do. True, the way some men operate it does their eyes no more harm than it would to hoe corn. They will while filling teeth, be talking about every thing, see every thing, and will laugh and sing all the while. Such operators will not injure their eyes. But the man that fixes his attention, and fixes his eyes sharply and keenly upou the point where he is working, and holds them there, will suffer fatigue of the eyes, if nothing more.

How are all these things to be remedied? What can be done for the eyes to assist them and preserve them for the longest possible period? When the eyes fail very manifestly, so that they can not be made available by ordinary glasses, then the operation of the dentist is gone—it is the end of the matter with him, so far as the practice of dentistry is concerned. How may the eyes be preserved in the best possible condition? This is a very important question to those who have eyes yet in all their strength and vigor; of more importance, if possible, to those whose eyes have begun to fail because of age, or anything else.

One of the best means of affording relief to the eye when fatigued, as has been suggested, is to surround it by such objects as are pleasant to look upon. This matter ought to be studied, and we ought to have essay upon essay, upon this subject, so that we would thoroughly understand it.

In reference to taking care of the eyes, by using glasses during the time of operating, I will say a few words. I recommended some years ago the employment of glasses by persons whose eyes had not begun to fail to relieve them of this fatigue, occasioned by their fixedness upon a small point. By the use of glasses, magnifying the objects upon which the eye must rest, there would be a much larger surface presented. But the objection occurred to me, that the convergence of the eyes might produce difficulties that would be equal to the advantage derived from this enlargement of the object, but I have not found it so. The convergence of the eyes to a certain extent will not injure the eyes. That is the principle given or the idea entertained, that if you use glasses you will shorten the vision, and the organ will get out of play; but I do not regard it as true, unless it is pressed to a greater extent than it need be. It is true in regard to the blacksmith's arm—as referred to last night—though it is not a parallel case—that he may by the use of the muscles of his arm increase its size and strength; but he may also exert the muscles to such an extent as to abuse them, and lose their strength, and the arm become less than before. But up to a legitimate and proper point there may be a convergence of the eyes without difficulty or injury. When there is danger of any difficulty in this way, it may be know as the eyes will complain, and cry out in such a manner that you will have to hear them. You will know it when there is a sense of pain with pressure, and fatigue on the part of the eyes. It will easily be recognized, and ought always to be regarded.

In regard to this matter of changing from action to rest, from work to rest, from one condition to another, much might be said. A horse will get along very well up hill and down hill, better than to travel continuously on a level road; change seems to be necessary. This principle as it applies to the eyes, is advisable, hence, I have resorted to this principle for the last few years. I have used glasses in such a way as to give frequent change to the eyes, changing the focus, and consequently changing the focus of the eyes, and some change as to the direction of the rays of light upon the nerve distribution in the eyes, by using different glasses with a different focus, and I have derived very considerable advantage in that way. I used two glasses, throwing them one over the other. Then after that it occurred to me, that perhaps a different arrangement might be made, so as to use a large magnifying glass over one eye, and not use the other at all. One of the eyes can be used with a strong magnifying glass without any apparent fatigue at all, putting a black dish over the other. That arrangement had some inconvenience, but I could work for a much longer period without fatigue to the eyes. Upon this same principle watch-makers use the same lens for years and years, a whole lifetime, over one eye without the eye suffering very materially. In order to carry out this principle more fully, I got an idea some years ago of using two glasses, the upper one having a focal distance of about eighteen or twenty inches, and the lower one of nine or ten inches. These I use in operating, one thrown upon the other (the speaker here showed with his glasses the manner in which he used them, explaining that they were so adjusted as that he could use either or both of them, or the unassisted eye as he wished.)

Thus you see I have three or four ranges of vision. In the first place I have the unassisted eye, by which I can see things on the table and about the room very distinctly, perhaps as well as I ever did. Coming to the operation I look through the lower glasses. In some cases, when I want to see if the operation is going on all right, I will look through the glasses thrown together; so I have here four grades of vision, and there seems to be that change from one grade of vision to the other that gives relief to the eyes, so that they do not suffer in any sense at all, as they formerly did when I used but one glass. This arrangement I find more convenient, and my eyes suffer less than with any other arrangement I ever had. I would part with almost anything else I have about me, in the way of my arrangements and appliances, rather than these glasses.

I will add in respect to this matter, that four years ago my sight was far less perfect than now, I can now read with the unassisted eye print or manuscript very well that three or four years ago, or two years ago, I could not read at all. I mention this to show that the use of glasses is not injurious.

Dr. Rosson—Do you believe it would have been better for your sight if you had commenced with that arrangement of glasses when you first began to use glasses?

Dr. Taft—I am very sure it would. When I commenced the use of glases I was broken down, so that I could operate but little, scarcely any for a year. My eyes were so weak I could not operate without glasses. I resorted to the glasses, and get along tolerably well, but suffered still in that respect; but three or four years ago, when I began the use of these double glasses, I did much better.

Those of you who have seen Dr. Atkinson operate know that he uses two glasses. He was driven to it simply by the necessity of the case, and by his appreciation of the difficulties, and his understanding of what was needed. If I had had something of this kind five or six years ago, I would not have suffered from this inability of eyesight as I did in the reading of print, manuscript, etc.

Dr. J. H. Warner—I would like to know whether you would recommend the use of glasses of any kind to young men who feel no symptoms of impaired vision.

Dr. Taft—My impression is that almost anybody could use them to advantage. If the eyes suffer fatigue, and you would use glasses arranged upon this principle, my impression is that the eyes would receive relief if you were but ten years old. Prolonged use of common glasses on the eyes may be injurious, but the eyesight of any person would doubtless receive advantage from the use of such glasses as these. You can readily understand the reason of this: Suppose a person of but ten years of age fixes his eyes upon a small point for a long time, the muscles of the eye must become strained and tired. Suppose the size of the object looked at is doubled, would it not relieve the eye from that strain? The relief afforded, it seems to me, is in that way merely by the expansion of the surface at which you look. The manner in which the light strikes the glasses will have a modifying influence; sometimes the light is thrown on the eyes in an oblique manner, which is quite unpleasant. In the arrangement of the glasses that must be guarded against, so that the rays will come as directly into the eye as possible.

Dr. Warner—I believe that to be the true theory, but I don't believe the glasses magnify the objects looked at enough to materially change the position of the muscles of the eye.

Dr. Taft—You say you don't believe the glasses magnify enough to amount to anything. A very little change gives relief many times. It is the constant straining and holding the eye to a very fine point, that wearies. And this refers not only to the muscles but to other parts in the structure of the eye. Other parts are in that strained condition as well as the muscles.

Dr. F. H. Rehwinkle—I merely wish to say that I believe the remarks of Dr. Warner are applicable where a person is short or near-sighted. In short-sighted persons, wearing glasses will not remedy the evil. In other words the ball of the eye becomes rounder through the use of glasses, while in long sighted persons it will increase the flatness of the eye-ball. It is a well-known fact that near-sighted persons, who commence wearing glasses in early youth, and wear them constantly, are obliged every few years, or every year or two to change to glasses having stronger and stronger magnifying power, whereas if they abstain as much as possible from their use, they may get along with the same glasses or many years. As people advance in age the pupil of the eye becomes more flattened. On the other hand in far-sighted persons, the pupil becomes more round. In cases where the use of glasses is persisted in too early, I can see there is danger of doing harm, but I don't think an occasional use of magnifyers for a change has any injurious effects upon the eye.

